# Sorghum stem aerenchyma formation is regulated by *SbNAC_D* during internode development

**DOI:** 10.1002/pld3.85

**Published:** 2018-11-12

**Authors:** Anna L. Casto, Brian A. McKinley, Ka Man Jasmine Yu, William L. Rooney, John E. Mullet

**Affiliations:** ^1^ Department of Biochemistry and Biophysics Texas A&M University College Station Texas; ^2^ Molecular and Environmental Plant Sciences Graduate Program Texas A&M University College Station Texas; ^3^ Biochemistry and Biophysics Graduate Program Texas A&M University College Station Texas; ^4^ Department of Soil and Crop Sciences Texas A&M University College Station Texas

**Keywords:** *D*‐gene, germplasm, programmed cell death, *Sorghum bicolor*

## Abstract

*Sorghum bicolor* is a drought‐resilient C4 grass used for production of grain, forage, sugar, and biomass. Sorghum genotypes capable of accumulating high levels of stem sucrose have solid stems that contain low levels of aerenchyma. The *D*‐locus on SBI06 modulates the extent of aerenchyma formation in sorghum stems and leaf midribs. A QTL aligned with this locus was identified and fine‐mapped in populations derived from BTx623*IS320c, BTx623*R07007, and BTx623*Standard broomcorn. Analysis of coding polymorphisms in the fine‐mapped *D*‐locus showed that genotypes that accumulate low levels of aerenchyma encode a truncated NAC transcription factor (Sobic.006G147400, *SbNAC_d1*), whereas parental lines that accumulate higher levels of stem aerenchyma encode full‐length NAC TFs (*SbNAC‐D*). During vegetative stem development, aerenchyma levels are low in nonelongated stem internodes, internode growing zones, and nodes. Aerenchyma levels increase in recently elongated internodes starting at the top of the internode near the center of the stem. *SbNAC_D* was expressed at low levels in nonelongated internodes and internode growing zones and at higher levels in regions of stem internodes that form aerenchyma. *SbXCP1*, a gene encoding a cysteine protease involved in programmed cell death, was induced in *SbNAC_D* genotypes in parallel with aerenchyma formation in sorghum stems but not in *SbNAC_d1* genotypes. Several sweet sorghum genotypes encode the recessive *SbNAC_d1* allele and have low levels of stem aerenchyma. Based on these results, we propose that *SbNAC_D* is the *D*‐gene identified by Hilton (1916) and that allelic variation in *SbNAC_D* modulates the extent of aerenchyma formation in sorghum stems.

## 1 INTRODUCTION

*Sorghum bicolor* is a highly productive, drought‐resilient C4 grass used for production of grain, forage, sugar, and biomass (Mullet et al., 2014; Rooney, 2004; Rooney, Blumenthal, Bean, & Mullet, 2007). Sorghum has a large and diverse germplasm useful for genetic analysis (Mace et al., 2009, 2013; Morris et al., 2013). The species is diploid and mostly inbreeding with a sequenced genome comprised of ten chromosomes that span ~800 Mbp (Mace et al., 2013; McCormick et al., 2018; Paterson et al., 2009). The sorghum research community has developed TILLING (Xin et al., 2008) and transcriptome resources (McCormick et al., 2018; Shakoor et al., 2014), and sorghum is amenable to transformation (Wu et al., 2014) and genome editing (Jiang et al., 2013). These genetic resources and attributes have facilitated the use of sorghum for hybrid breeding, comparative genomics, and the discovery of numerous important sorghum genes using QTL‐assisted gene identification (Hilley, Truong, Olson, Morishige, & Mullet, 2016; Hilley et al., 2017; Klein et al., 2005; Lin et al., 2012; Murphy et al., 2011, 2014).

Plant stems are essential for the transport of water and nutrients to and from roots and leaves. Stems also serve as a temporary storage depot for excess photosynthate and nutrients not immediately needed for basic metabolism, growth, or grain filling (Slewinski, 2012). In grasses, internode numbers and lengths impact canopy architecture, radiation use efficiency, nitrogen, and water use (George‐Jaeggli, Jordan, van Oosterom, Broad, & Hammer, 2013; Mullet et al., 2014). High‐biomass sorghums develop 4‐ to 5‐m‐length stems that can account for up to 84% of harvested biomass (Olson et al., 2012). Sorghum stems are comprised of phytomers produced approximately every 4 days by the shoot apical meristem (Kebrom, McKinley, & Mullet, 2017). Stem internode elongation is repressed during the juvenile phase but increases during the vegetative phase (Kebrom et al., 2017) or following floral initiation (Quinby, 1974). Several genes that regulate sorghum stem growth have been identified including an ABCB1 auxin transporter encoded by *Dw3* (Multani et al., 2003), a plasma membrane protein encoded by *Dw1* (Hilley et al., 2016; Yamaguchi et al., 2016) that is involved in brassinosteroid signaling and cell proliferation (Hirano et al., 2017), and an AGC kinase encoded by *Dw2* (Hilley et al., 2017).

Sorghum stems have the capacity to accumulate high concentrations (~0.5 M) and large amounts of sucrose (up to 40% by weight) similar to sugarcane (Bihmidine, Hunter, Johns, Koch, & Braun, 2013; Godoy & Tesso, 2013; Gutjahr et al., 2013; Lingle, 1987; McKinley, Rooney, Wilkerson, & Mullet, 2016). Stem volume, juiciness, and sugar concentration have been under selection in sweet sorghum in order to maximize sugar yield (Burks, Kaiser, Hawkins, & Brown, 2015; Carvalho & Rooney, 2017; Murray et al., 2008). Traits and QTL that modulate the accumulation of stem sugars have been identified (Burks et al., 2015; Felderhoff et al., 2012; Guan et al., 2011; Han et al., 2015; Hart, Schertz, Peng, & Syed, 2001; Mocoeur et al., 2015; Murray et al., 2008; Shiringani, Frisch, & Friedt, 2010; Srinivas et al., 2009; Xiao‐ping, Jin‐feng, Cui‐ping, & Acharya, 2011), and the biochemical and molecular basis of stem sugar accumulation has been investigated (Calviño, Bruggmann, & Messing, 2008; Gutjahr et al., 2013; Lingle, 1987; McKinley et al., 2016; Milne et al., 2017). The stems of sorghum genotypes that accumulate high levels of sucrose generally lack or accumulate low levels of aerenchyma and maintain functional pith parenchyma with large vacuoles where sucrose is sequestered (Burks et al., 2015; Carvalho & Rooney, 2017; Hilson, 1916). Other sorghum genotypes have stems that convert pith parenchyma into aerenchyma to varying degrees (Carvalho & Rooney, 2017; Hilson, 1916; Stephens & Quinby, 1939).

Genetic variation in the extent of aerenchyma formation in stems and leaf midribs was noted ~100 years ago (Hilson, 1916). Genetic analysis of variation in stem juiciness (vs. pithiness) and the color of leaf midribs (green vs. white) revealed that in some populations, the genetics of aerenchyma formation was simple and that a single dominant gene largely accounted for the appearance of “pithy” stems and white midribs caused by accumulation of aerenchyma (Ayyangar, Ayyare, Rao, & Nambiar, 1936; Hilson, 1916). Swanson and Parker assigned the symbols *D* and *d* to this main effect genetic factor for stem and leaf midrib “dryness” (Swanson & Parker, 1931). Genotypes with pithy stems and white midribs encode an active *D*‐allele that causes the accumulation of aerenchyma in stems and leaf midribs (Hilson, 1916; Stephens & Quinby, 1939). A major QTL corresponding to the *D*‐gene was mapped to LG‐06 by Xu et al. (2000), a location subsequently confirmed using other populations (Hart et al., 2001; Srinivas et al., 2009) and by GWAS (Burks et al., 2015). A stem moisture QTL was also mapped to the same location on LG‐06 (Han et al., 2015).

Flooding and hypoxia induce formation of aerenchyma in sorghum roots (Drew, Jackson, & Giffard, 1979). Hypoxia increases the synthesis and accumulation of ethylene in roots, and ethylene induces programmed cell death‐mediated root aerenchyma formation (Drew, He, & Morgan, 2000). In species such as rice, some root aerenchyma form in the absence of flooding and additional aerenchyma form in response to flooding or ethylene treatment (Yamauchi, Shimamura, Nakazono, & Mochizuki, 2013). In maize, accumulation of aerenchyma is induced by hypoxia (Drew et al., 2000; Yamauchi et al., 2016). In contrast, the wetland species *Juncus effusus* form root aerenchyma in the absence of ethylene and flooding (Visser & Bogemann, 2006). Programmed cell death is also induced in response to pathogens through the hypersensitive response (Liang et al., 2003). Moreover, programmed cell death occurs in many tissues such as root caps (Fendrych et al., 2014), xylem (Pesquet et al., 2013), the endosperm (Uauy, Distelfeld, Fahima, Blechl, & Dubcovsky, 2006; Young & Gallie, 2000), and during emergence of adventitious roots (Mergemann & Sauter, 2000) and organ senescence (Kim et al., 2014). The molecular events and genes involved in developmental and abiotic stress‐induced programmed cell death have been identified in a number of plants (De Clercq et al., 2013; Rajhi et al., 2011; Takahashi, Yamauchi, Rajhi, Nishizawa, & Nakazono, 2015; Yamauchi et al., 2017).

The yield and composition of stem biomass are of critical importance for high‐biomass sorghum since stems account for ~80% of the harvested biomass (Olson et al., 2012). Variation in stem aerenchyma formation can impact stem biomass yield and composition by altering the capacity of stems to accumulate sucrose. Therefore, in this study we characterized when and where stem aerenchyma form during stem and internode development and cloned the sorghum *D*‐gene to better understand the genetic network that modulates this important stem trait. The analysis showed that the *D*‐gene encodes a NAC transcription factor expressed in stems that regulates the expression of a gene involved in developmental programmed cell death and the formation of aerenchyma.

## 2 MATERIALS AND METHODS

### 2.1 Plant materials and growing conditions

All plants were grown in a glasshouse under 14‐hr long days in 14.5‐L pots with MetroMix 900 (Sun Gro Horticulture) fertilized as needed with Peters 20‐20‐20. Plants were thinned to one plant per pot and grown at 10–20 cm spacing. Seed for all genotypes and populations was obtained from the Texas A&M Sorghum Breeding Program (College Station, TX).

### 2.2 Plant phenotyping and visualization of aerenchyma by microscopy and CT scanning

The extent of aerenchyma (AER) formation was rated by visual inspection of cross sections of internodes taken approximately at the midpoint between nodes. Ratings were based on a scale from 1 to 5 (1, no AER; 5, high AER) in a manner similar to prior studies (Supporting Information Figure S1) (Carvalho & Rooney, 2017; Nilsen et al., 2017). Results from visual rating were similar to ratings based on a percent of area calculation when data were used for QTL analysis (Carvalho & Rooney, 2017). All AER ratings are the mean of at least three biological replicates unless otherwise indicated.

Multi‐slice computed tomography (MSCT) scans of five R07020 stems were collected at the Diagnostic Imaging & Cancer Treatment Center of the Texas A&M Veterinary Medicine & Biomedical Sciences facilities in College Station, Texas. A SOMATOM Definition AS+ (SIEMENS) was used with settings of 120 kVp, 1.024 pixels/mm, at a 0.6 mm slice thickness (Gomez, Carvalho, Shi, Muliana, & Rooney, 2018).

For microscopic imaging, hand sections were made of representative R07020 internodes at different stages of internode development to investigate changes in the distribution and extent of aerenchyma accumulation in stems. The sections were imaged under white light using a Zeiss M^2^Bio Fluorescence Combination Zoom Stereo/Compound Microscope coupled with a Zeiss AxioCam digital camera (Kramer Scientific). Photographs were captured using the Zeiss AxioVision 3.0.6 software.

### 2.3 Quantitative trait locus (QTL) analysis and fine‐mapping

Stem aerenchyma QTL were mapped in three populations: BTx623 x IS3620c (RILs, *n* = 380), BTx623 x R07007 (F5, *n* = 215), and BTx623 x Standard Broomcorn (F2, *n* = 133). All plants were grown to anthesis and phenotyped by cutting the internode below the peduncle at a midpoint between the upper and lower node. The cross sections were visually inspected and rated on a scale from 1 (no aerenchyma) to 5 (high aerenchyma) (Supporting Information Figure S1). One plant of each RIL from the BTx623*IS3620c population was grown in the summer of 2014 under long days. Three plants of each line of the BTx623*R07007 population were grown in a glasshouse in the spring of 2015 under long days. Genotyping and genetic map construction were performed as described in Truong, McCormick, Morishige, and Mullet (2014), and DG marker sequences were mapped to version 3.1 of the sorghum reference genome assembly (*Sorghum bicolor* v3.1 DOE‐JGI, http://phytozome.jgi.doe.gov/), using BWA (Li & Durbin, 2009). INDEL realignment and joint variant calling were performed with the GATK using the naïve pipeline of the RIG workflow (McCormick et al., 2018). QTL mapping was performed in R/qtl using composite interval mapping (CIM) with 1,000 permutations and *α* = 0.05 (Broman, Wu, Sen, & Churchill, 2003). BTx623*IS3620c and BTx623*R07007 populations were mapped as RILs, while BTx623*Standard Broomcorn was mapped as an F_2_. The BTx623*IS3620c population was used to fine‐map *SbNAC_D*. Five plants from each line that had recombination breakpoints in the 2‐LOD region spanning the main effect QTL on SBI‐06 were grown to anthesis and phenotyped to confirm aerenchyma ratings.

### 2.4 DNA sequencing and sequence analysis

Coding region sequence variants between the four parental lines used for QTL analysis were obtained from publicly available whole‐genome sequences (Phytozome v12.1.6). The effect of protein sequence variants was analyzed using PROVEAN (Choi, Sims, Murphy, Miller, & Chan, 2012). Genomic DNA was extracted from leaf tissue of diverse sorghum lines according to the Quick‐DNA™ Plant/Seed Kit (Zymo Research) instructions. *SbNAC_D* was amplified by polymerase chain reaction (PCR) and was sequenced by Sanger sequencing. All template amplification and sequencing primers are listed in Supporting Information Table S6.

### 2.5 Protein sequence analysis

Protein sequences of the closest homologs of *SbNAC_D* in grass species with available genome sequences were identified using BLAST (Phytozome v12). To identify the sugarcane homolog of *SbNAC_D*, the sorghum cDNA sequence was used in a BLAST search of the sugarcane transcriptome (SUCEST‐FUN Database, http://sucest-fun.org). Protein sequences were aligned using MUSCLE (Edgar, 2004) and visualized using Jalview (Waterhouse, Procter, Martin, Clamp, & Barton, 2009). Evolutionary trees were inferred using the neighbor‐joining method (Jones, Taylor, & Thornton, 1992) in MEGA7 (Kumar, Stecher, & Tamura, 2016). All positions containing gaps and missing data were eliminated.

### 2.6 cDNA sequencing and qRT‐PCR

Total RNA was extracted from all samples using the Direct‐zol™ RNA Miniprep Kit (Zymo Research), and cDNA was synthesized using SuperScript™ III First‐Strand Synthesis SuperMix for qRT‐PCR (Thermo Fisher Scientific). For analysis of gene expression in *SbNAC_D* and *SbNAC_d1* RILs, the internode subtending the peduncle was collected before aerenchyma formation and 7 – 10 days later when aerenchyma were visible. Internodes were collected from three biological replicates. For the developmental analysis of gene expression in R07020, internodes were collected from three biological replicates at midday. Tissue was collected from the two short nonelongated internodes immediately below the shoot apex that were approximately 1–2 cm in length (int1, int2). Internodes 3 and 4 (int3‐X, int4‐X) were subsectioned into 1‐cm sections. For the fully elongated internodes 5–7 (int5‐X, int6‐X, int7‐X), 2‐cm sections were isolated from the top and base of each internodes for analysis. A list of gene targets and primers used for the analyses is provided in Supporting Information Table S6. For all qRT‐PCR experiments, relative expression was determined using the comparative cycle threshold (*C*_t_) method. Raw *C*_t_ values for each sample were normalized to *C*_t_ values for the reference gene *SbUBC* (Sobic.001G526600). Reference gene stability across all samples was determined by NormFinder (Andersen, Jensen, & Ørntoft, 2004). ΔΔ*C*_t_ values were calculated relative to the sample with the highest expression (lowest Ct value). Relative expression values were calculated with the $2^{-\Delta \Delta C_{t}}$ method (Pfaffl, 2001). Fold change in gene expression was calculated based on Δ*C*_t_ values between the samples with the lowest and highest expression according to the equation $\text{FC}=2^{\left(\Delta C_{t}\left(\max\right)-\Delta C_{t}\left(\min\right)\right)}$. Primer specificity was tested by dissociation curve analysis and gel electrophoresis.

For cDNA sequencing, total RNA was extracted from the leaf sheaths of BTx623 and IS3620c and used for cDNA synthesis as described above. The cDNA corresponding to *SbNAC_D* was amplified and sequenced using Sanger sequencing. The primers used for template amplification and sequencing are listed in Supporting Information Table S5.

### 2.7 Statistical analyses

All statistical analyses were performed in GraphPad Prism, version 7, for Windows (GraphPad Software, La Jolla California USA, www.graphpad.com).

## 3 RESULTS

### 3.1 Sorghum genotypes vary in stem aerenchyma accumulation

**Figure 1:**
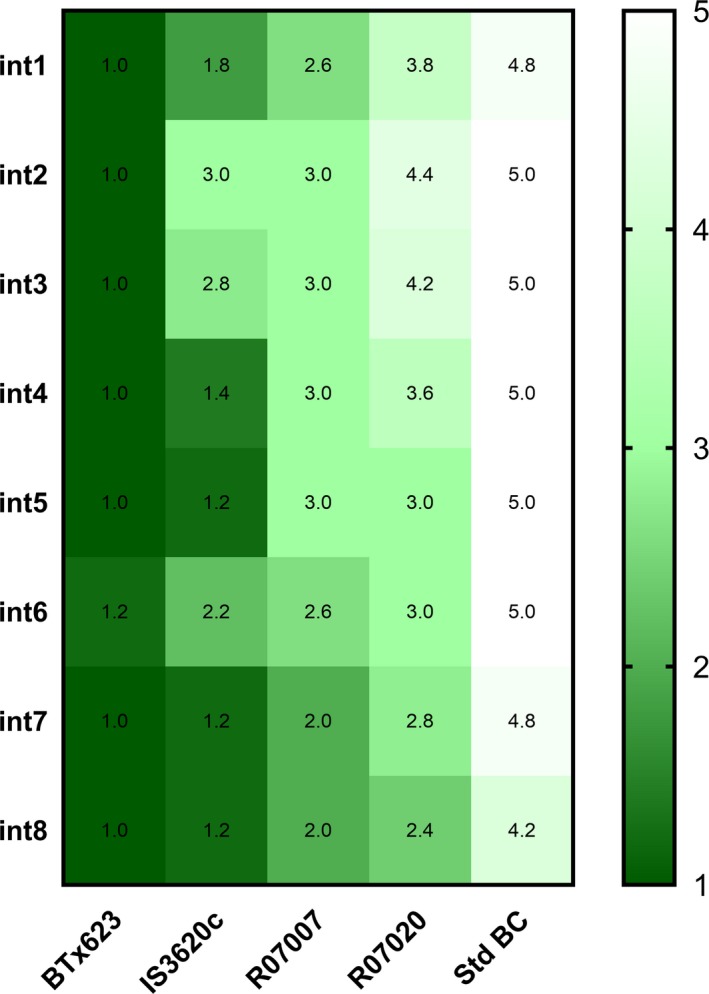
Variation in the extent of stem aerenchyma (AER) in diverse sorghum genotypes. Internodes of five sorghum genotypes were screened for the extent of AER formation in eight fully elongated internodes using a scale from 1 (minimal AER, dark green) to 5 (maximum AER, white) (heat map scale to the right). Internode 1 (int1) is the internode at the top of the stem below the peduncle. BTx623, an elite grain line, showed low levels of AER formation in all internodes. Standard Broomcorn (Std BC) had the highest levels of AER across all internodes among all screened genotypes. AER ratings are the average of at least three biological replicates

Sorghum genotypes were grown in a glasshouse and screened for variation in the extent of stem aerenchyma accumulation in order to identify genotypes for further study and parental lines of populations for QTL‐assisted identification of genes involved in stem aerenchyma formation. The extent of aerenchyma formation was rated by visual inspection of stem cross sections taken mid‐internode on a scale from 1 (no aerenchyma) to 5 (high aerenchyma) (Supporting Information Figure S1). This screen showed that the stems of BTx623 contained low levels of aerenchyma, whereas the stems of IS3620c, R07007, R07020, and Standard broomcorn had higher levels of stem aerenchyma (Figure 1). For most genotypes, aerenchyma levels were similar in fully elongated internodes along the length of the stem. IS3620c was more variable and showed a trend toward higher aerenchyma in the internodes immediately below the peduncle (Int1‐3). Standard broomcorn stems contained the highest levels of stem aerenchyma among the genotypes screened.

### 3.2 QTL‐assisted identification of the *D*‐gene

**Figure 2:**
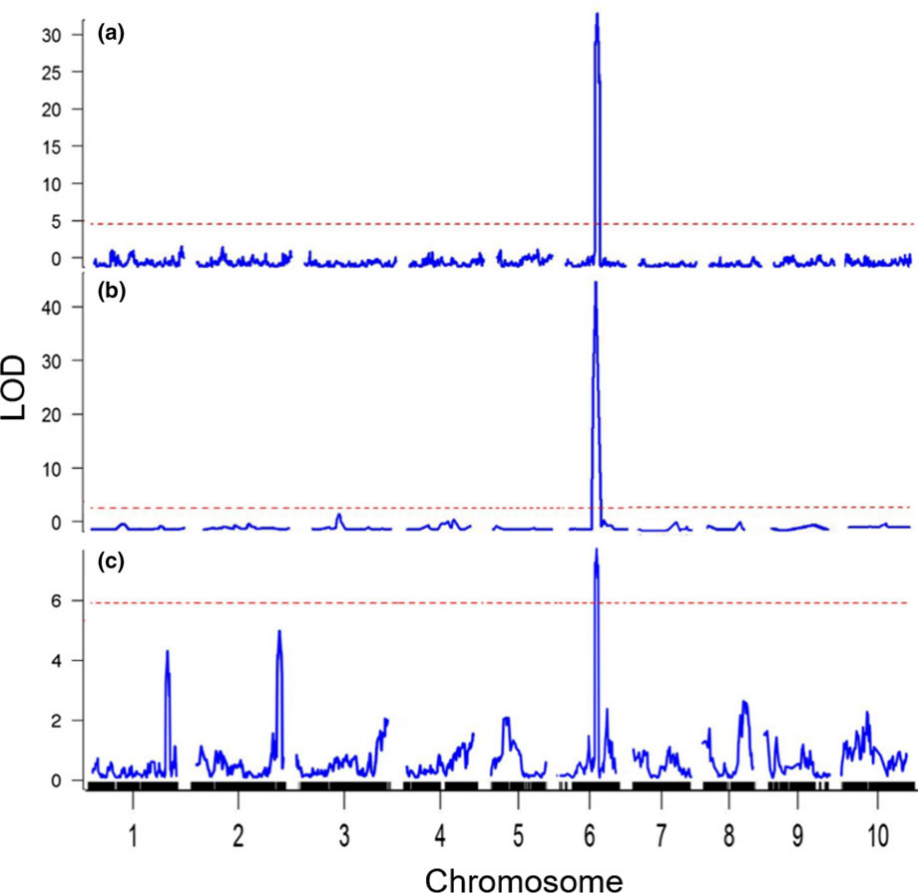
QTL associated with variation in aerenchyma (AER) formation were mapped in three biparental populations: BTx623*IS3620c (a, *n* = 380, RIL), BTx623*R07007 (b, *n* = 215, F5), and BTx623*Standard Broomcorn (c, *n* = 133, F2). The extent of AER formation in each line was rated visually in cross sections at a midpoint of fully elongated internodes. Main effect QTL colocalized to chromosome 6 in all three populations where the BTx623 allele reduced stem AER formation. QTL analyses were performed in R/qtl using composite interval mapping. AER ratings in BTx623*IS3620c were from one biological replicate per RIL. AER ratings in BTx623*R07007 are the average of three biological replicates per individual

The genetic network that regulates the extent of stem aerenchyma accumulation was investigated using QTL analysis. The results of genotype screening (Figure 1) indicated that populations derived from crosses involving BTx623*IS3620c, BTx623*R07007, and BTx623*Standard broomcorn would be useful for mapping QTL for variation in stem aerenchyma accumulation. Populations derived from each of these crosses were grown in a glasshouse and scored for variation in stem aerenchyma accumulation in internodes of fully elongated stem internodes. The stem aerenchyma phenotypes and pre‐existing genetic maps for these populations (Hilley et al., 2016, 2017; Truong et al., 2014) were used to identify QTL that affect the extent of aerenchyma formation in stems. A main effect QTL that modulates variation in stem aerenchyma accumulation was identified on SBI06 at approximately 50 Mbp in all three populations (Figure 2). The LOD scores of the QTL on SBI06 were >20 in the BTx623*IS3620c and BTx623*R07007 RIL populations and ~7.4 in the BTx623*Standard broomcorn F2 population (Supporting Information Table S1). Two additional QTL with subthreshold LOD scores located on SBI‐01 and SBI‐02, respectively, were detected using the Standard broomcorn population (Figure 2c). In all three populations, lines with homozygous BTx623 haplotypes spanning the QTL on SBI06 had low stem aerenchyma ratings and lines lacking BTx623 haplotypes accumulated the higher levels of stem aerenchyma (Supporting Information Figure S2).

**Figure 3:**
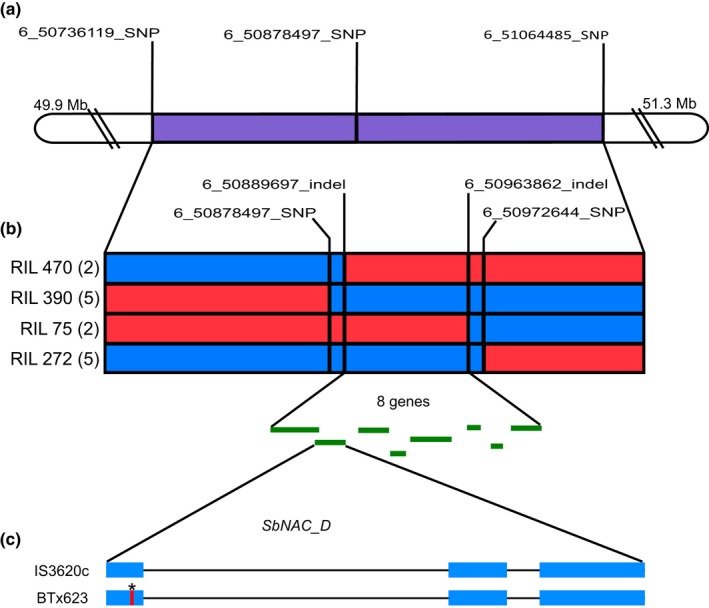
Positional cloning of SbNAC_D. (a) The marker 6_50878497_SNP had the highest LOD score in the QTL interval and was used to define a 2‐LOD interval spanning the QTL (purple). (b) RILs from BTx623*IS3620c (RIL 470, RIL 390, RIL 75, RIL 272) with breakpoints within the 2‐LOD interval were identified and phenotyped for the AER formation at anthesis. AER ratings are indicated in parentheses next to the RIL label to the left of the diagram. Blue haplotypes correspond to IS3620c alleles (+AER), and red haplotypes correspond to BTx623 alleles (−AER). The fine‐mapped interval contained eight genes. (c) Sobic.006G147400 (SbNAC_D) was identified as the best candidate for the “D” gene. The BTx623 allele contains a T‐to‐C polymorphism that causes a Gln to STOP (Q39X) change in the first exon of BTx623 (red line)

The QTL located on SBI06 that modulates stem aerenchyma accumulation in the current study is aligned with a QTL on LG‐06 previously identified as encoding the *D*‐gene (Hart et al., 2001). The prior study was based on the analysis of 132 RILs derived from BTx623*IS3620c and a low‐resolution genetic map. To fine‐map the *D*‐locus, the current study utilized 380 RILs derived from BTx623*IS3620c (Burow et al., 2011) and a high‐resolution genetic map constructed by scoring the segregation of ~10,091 DG markers in the expanded RIL population (Truong et al., 2014). A SNP marker located at 50,878,497 on SBI06 was tightly linked to the aerenchyma QTL (Figure 3a). Four RILs with breakpoints immediately flanking this stem aerenchyma QTL marker on SBI06 were identified (Figure 3b). SNP and INDEL markers useful for further fine‐mapping breakpoints within the *D*‐locus in the four RILs were identified from an alignment of the IS3620c whole‐genome sequence and the BTx623 reference sequence (Figure 3b) (McCormick et al., 2018). Sequence analysis of the markers in the four RILs delimited the *D*‐locus to a 73‐kbp region (Figure 3c, Supporting Information Table S2). Based on gene annotation information in Phytozome (Sbiv3.1), the 73‐kbp region encodes a NAC (NAM, ATAF1/2, CUC) transcription factor, three genes encoding threonine aldolase, a MYB‐like DNA‐binding factor, and three genes encoding predicted (or unknown) proteins that lack functional annotation (Supporting Information Table S2).

**Figure 4:**
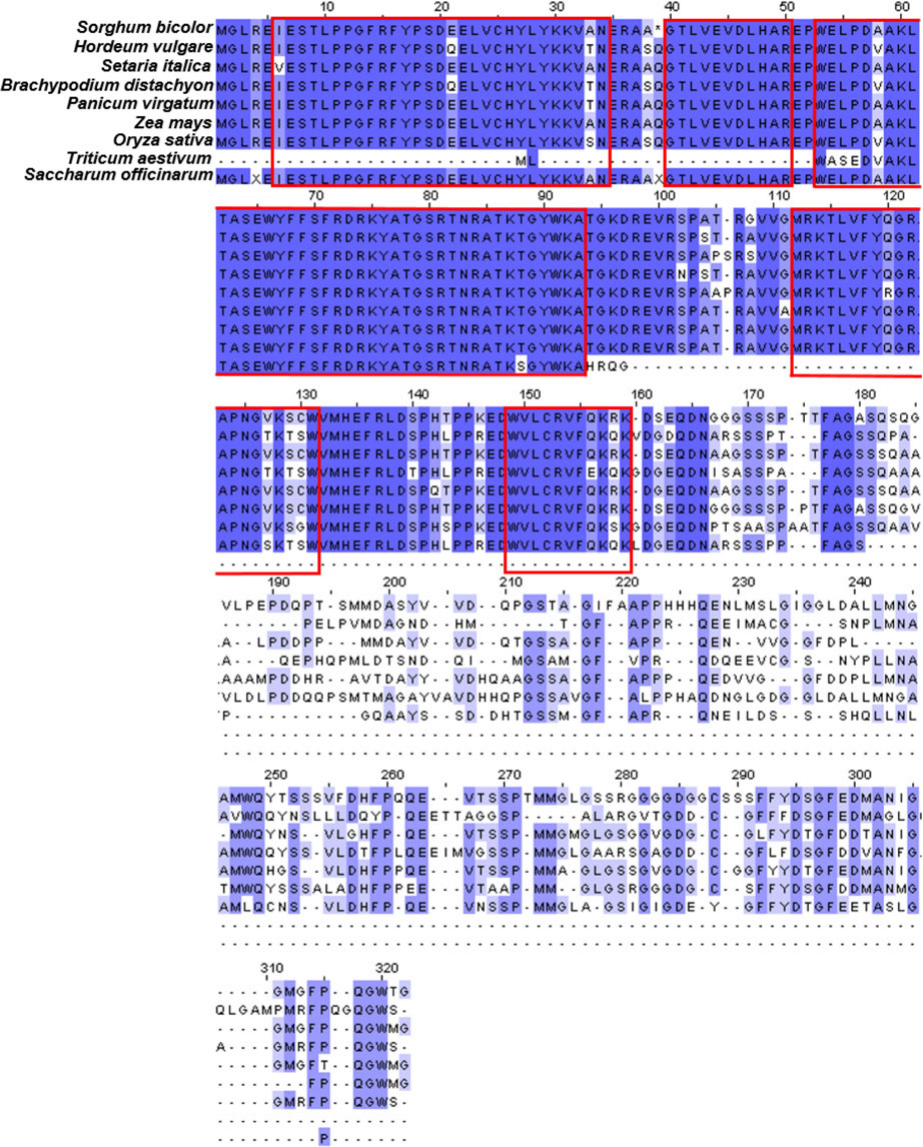
Protein sequence alignment of SbNAC_D homologs in select grass species. N‐terminal sequences (1–170) of the SbNAC_D homologs are highly conserved, including protein motifs conserved in NAC transcription factors (highlighted by red boxes). Protein sequences were aligned using MUSCLE (Edgar, 2004) and visualized using Jalview (Waterhouse et al., 2009)

The genes in the delimited *D*‐locus were analyzed for the presence of sequence variants in coding regions to help identify candidate genes that could correspond to the *D*‐gene. Gene intron/exon annotations were checked using RNAseq data (Phytozome v12), and most of the gene annotations were supported by this analysis (McCormick et al., 2018). However, Sobic.006G147400 was annotated with three introns although RNAseq data and information from direct cDNA sequencing (Supporting Information Figure S3) provided evidence for only the two larger introns. Moreover, the incorrectly annotated intron in Sobic.006G147400 encodes amino acid sequences (V32 – E44) that are present in the sorghum cDNA and in homologs of this gene (Figure 4). Therefore, an annotation of Sobic.006G147400 that contains two introns was used for subsequent analysis of coding variants. A proposed intron in Sobic.006G147800 was also not well supported by RNAseq data possibly because expression of this gene was low resulting in incomplete coverage of the gene (Phytozome v12). A gene annotation lacking introns resulted in a continuous coding region through the predicted intron consistent with the annotation of a homolog of Sobic.006G147800 in Setaria that lacks an intron (Phytozome). Based on these results, the annotation of Sobic.006G147800 with and without the proposed intron was analyzed for coding variants.

Genetic analysis in the three populations is consistent with the prediction that there is a sequence variant (or variants) in BTx623 within the *D*‐locus that inactivates a gene or regulatory element that normally increases stem aerenchyma formation and that this sequence variant is not present in IS3620c, R07007, and Standard broomcorn. Polymorphisms were identified in the coding regions of the eight genes within the delimited *D*‐locus that distinguish the parental lines of the QTL mapping populations (BTx623, IS3620c, R07007, and Standard broomcorn). In addition, coding variants were identified in Rio and SC170, genotypes that have low levels of stem aerenchyma similar to BTx623 (see below and Table 1). Coding variants were further analyzed to determine whether they are predicted to alter protein function (Supporting Information Table S3) (Choi et al., 2012). This analysis showed that three genes in the delimited *D*‐locus lacked deleterious coding polymorphisms (Sobic.006g147450, Sobic.006g147500, Sobic.006g148000) that distinguished BTx623, Rio, SC1470, IS3620c, R07007, and Standard broomcorn (Supporting Information Table S3). However, the analysis of coding variants identified a sequence variant in the low aerenchyma lines BTx623, Rio, and SC170 that creates a stop codon (Q39X) in the NAC transcription factor encoded by Sobic.006g147400 (Supporting Information Table S3). In contrast, genotypes that accumulate higher levels of stem aerenchyma (IS3620c, R07007, and Standard broomcorn) encoded full‐length NAC proteins. The stop codon (Q39X) in Sobic.006G147400 present in BTx623, Rio, and SC170 removes motifs B‐D of the NAC domain (Zhu, Nevo, Sun, & Peng, 2012) and the rest of the C‐terminal portion of the protein. Sobic.006G147600, a gene that encodes threonine aldolase, contained a change in amino acid sequence (S218P) in IS3620c and SC170 that was predicted to cause a deleterious change in protein function by PROVEAN analysis (Choi et al., 2012). SC170 has low aerenchyma, while IS3620c has high aerenchyma, so this deleterious change in amino acid sequence is unlikely to be associated with variation aerenchyma formation (Table 1). Two other sequence variants in Sobic.006G147700 present only in Standard broomcorn were predicted to cause deleterious changes in protein sequence. Sequence variants in Sobic.006G147800 were predicted to cause deleterious changes in protein function in R07007 and Standard broomcorn, but these changes were also present in Rio and SC170. Additionally, no coding region mutations in Sobic.006G147800 were identified in IS3620c, so variation coding variants in Sobic.006G147800 were not associated with the stem aerenchyma QTL in the population derived from BTx623*IS3620c. In summary, the analysis identified Sobic.006G147400 (NAC transcription factor) as the only gene with a deleterious coding variant in the low aerenchyma lines (BTx623, Rio, and SC170) that was not present in lines that accumulate higher levels of stem aerenchyma. Taken together, the analysis of coding variants indicated that Sobic.006g147400, a gene encoding a NAC transcription factor, contained a stop codon in BTx623, Rio, and SC170 that was not present in IS3620c, R07007, and Standard broomcorn, consistent with genetic prediction that BTx623 contains a mutation in a gene that is involved in induction of stem aerenchyma formation. As such, we designated the two alleles of this gene as *SbNAC_D* and *SbNAC_d1* (the allele in BTx623) to distinguish this gene from other *NAC*‐genes in sorghum.

**Table 1:** *SbNAC_D* alleles identified in various genotypes of *Sorghum bicolor*

Genotype	Allele	Amino acid change	AER rating
R07007	*SbNAC_D*	–	3–5^b^
R07020	*SbNAC_D*	–	4–5
IS3620c^a^	*SbNAC_D*	–	3–5
Standard broomcorn	*SbNAC_D*	–	5
Feterita	*SbNAC_D*	–	3–4
TX08001 (BTx623/R07007)	*SbNAC_D/d1*	–	3
BTx623^a^	*SbNAC_d1*	Q39X	1–2
Blackhull Kafir	*SbNAC_d1*	Q39X	1–2
Tx3197	*SbNAC_d1*	Q39X	1–2^b^
RTx430	*SbNAC_d1*	Q39X	1
SC170	*SbNAC_d1*	Q39X	1
Hegari	*SbNAC_d1*	Q39X	1
Sweet sorghums			
Rio	*SbNAC_d1*	Q39X	1–2^b^
Wray	*SbNAC_d1*	Q39X	1
Della	*SbNAC_d1*	Q39X	1–2^b^
Collier	*SbNAC_d1*	Q39X	1–2
Grassl	*SbNAC_d1*	Q39X	1

Notes

Aerenchyma (AER) ratings were scored on a scale from 1 to 5 (1, no AER; 5, high AER) at a midpoint of an internode. Ratings are an average of at least three biological replicates.

^a^Allele sequence was determined by whole‐genome sequences. All others were determined by Sanger sequencing. ^b^AER ratings were adapted from Carvalho & Rooney, 2017.

### 3.3 *D*‐alleles in sorghum germplasm

Sequence analysis of Sobic.006g147400 from R07007, R07020, Standard broomcorn, and Feterita showed that these genotypes encode *SbNAC_D*, consistent with the accumulation of relatively high levels of stem aerenchyma in the stems of each genotype (Table 1). The energy sorghum hybrid, TX08001, derived by crossing A/BTx623*R07007 and therefore heterozygous for *NAC_D* alleles (*NAC_D:NAC_d1*) had an intermediate aerenchyma rating (Table 1). The recessive *SbNAC_d1* allele found in BTx623 was also present in other lines used in grain sorghum breeding including Blackhull Kafir, Tx3197, RTx430, SC170, and Hegari, genotypes that accumulate relatively low levels of aerenchyma (Table 1). The sweet sorghums Rio, Della, Wray, Collier, and Grassl, genotypes that accumulate low levels of stem aerenchyma, also encode *SbNAC_d1* (Table 1). In this limited survey of sorghum germplasm, the presence of *SbNAC_D* in genotypes was correlated with high stem aerenchyma and *SbNAC_d1* with lower levels of stem aerenchyma.

### 3.4 Phylogenetic analysis of *SbNAC_D*

NAC transcription factors were named for three genes initially discovered to contain NAC domains that contain N‐terminal conserved motifs: NAM (no apical meristem), ATAF1 and ‐2, and CUC (cup‐shaped cotyledon) (Souer, van Houwelingen, Kloos, Mol, & Koes, 1996; Zhu et al., 2012). NAC transcription factors are unique to plants, and most plant species including sorghum encode >100 different NAC family members (Kadier et al., 2017). Phylogenetic analysis of grass genes encoding the *SbNAC‐D*‐like factors generated C4 grass and C3 grass groups (Supporting Information Figure S4). Protein alignment showed that the grass *NAC_D*‐like proteins were highly conserved across their N‐terminal 170 amino acids that span the NAC domain motifs A‐E (Figure 4). Greater divergence was observed across the C‐terminal portion of the grass *NAC_D*‐like proteins consistent with what has been found for other NAC proteins. The large N‐terminal deletion present in a *NAC_D* homolog of wheat may be a consequence of selection for solid stems in this grain crop (Nilsen et al., 2017). A *NAC_D* homolog of sugarcane was also truncated, which may contribute to reduced aerenchyma formation in stems of sugarcane (Figure 4).

*NAC* family genes related to *SbNAC_D* were identified in sorghum (Supporting Information Figure S5A) and other plants (Supporting Information Figure S5B) to see whether this would provide information about *SbNAC_D* function. The most closely related *NAC*‐gene family member in Arabidopsis, *AtNAC074* (At4G28530), is differentially expressed to some extent in xylem compared to phloem cambium (Zhao, Craig, Petzold, Dickerman, & Beers, 2005). NAC factors such as VND6/7, SND1, and NST1/2 play key roles in secondary cell wall formation and xylem differentiation, developmental processes that involve programmed cell death (PCD) (Nakano, Yamaguchi, Endo, Rejab, & Ohtani, 2015). *NAC*‐genes also play a role in PCD‐associated senescence (Kim et al., 2014). Therefore, sorghum *NAC*‐gene homologs of *NAC*‐genes involved in secondary cell wall formation and senescence were identified and compared with *SbNAC_D* homologs from grasses (Supporting Information Figure S6). This showed that grass *NAC_D‐like* genes grouped in a cluster distinct from *NAC*‐genes involved in secondary cell wall formation and senescence. The analysis also showed that *SbNAC_D* was closely related to ZmNAC22 (GRMZM2G081930), a member of the ZmNAC‐h (*NAC1*) subfamily (Peng et al. 2015). This subfamily is distinct from the ZmNAC‐j (VND6) and ZmNAC‐I (SND/NST1) subfamilies involved in secondary cell wall formation. These results indicate that the function of *SbNAC_D* differs from *NAC* transcription factors involved in xylem secondary cell wall formation/xylem differentiation and senescence.

### 3.5 Expression of *D*‐locus genes in RILs with BTx623 or IS3620c haplotypes

Sobic.006G147400 was identified as the best candidate gene for the *D*‐gene among the genes located in the *D*‐locus based on an analysis of coding sequence variants. However, it is possible that there are sequence variants among the parental genotypes used for QTL analysis that modify noncoding regulatory elements in the *D*‐locus that affect aerenchyma formation by altering the expression of one or more of the eight genes in the delimited *D*‐locus. To check this possibility, the expression of the genes in the delimited *D*‐locus was analyzed by qRT‐PCR using RNA from stems of three pairs of RILs derived from BTx623*IS3620c with similar flowering times and dwarfing genotypes that either encoded the IS3620c *D*‐locus haplotype or the BTx623 *d1*‐locus haplotype. RNA was extracted from the top internode of the stem from each RIL shortly after full internode elongation for qRT‐PCR analysis. The analysis showed no significant difference in expression of the 8 genes in the three pairs of RILs indicating that polymorphisms distinguishing BTx623 (*d1*) and IS3620c (*D*) in noncoding regions of the *D*‐locus were not altering the expression of genes in the *D*‐locus in this tissue (data not shown).

### 3.6 Developmental timing of stem aerenchyma accumulation and *SbNAC_D* expression

**Figure 5:**
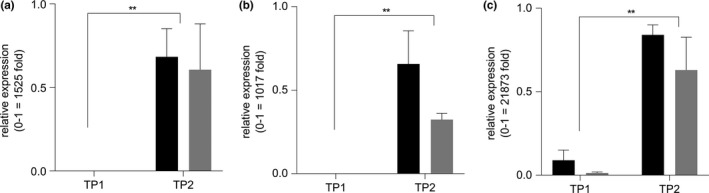
*SbNAC_D* expression in three pairs of *SbNAC_D (black bars)/SbNAC_d1 (grey bars)* RILs: (a) RIL226/RIL212, (b) RIL119/RIL474, and (c) RIL392/RIL49. Expression was analyzed in the internode subtending the peduncle via qRT‐PCR at time points before aerenchyma (AER) formation (TP1) and 7–10 days later when AER were visible in the internode (TP2). In all three RIL pairs, expression of *SbNAC_D* increased in parallel with AER formation. All gene expression was normalized to the expression of *SbUBC* in each sample. Relative expression was calculated via the $2^{-\Delta \Delta C_{t}}$ method relative to the internode section with the highest expression of the gene in each RIL pair (Pfaffl et al., 2001). Fold change in expression between the minimum and maximum values on the *y*‐axis was calculated based on *SbUBC* normalized values according to $\text{FC}=2^{\Delta C_{t}\left(\max\right)-\Delta C_{t}\left(\min\right)}$. Expression values are the average of three biological replicates. Differences in expression values between TP1 and TP2 were analyzed by ANOVA in GraphPad Prism v7. **, *p* < 0.005

During the analysis above, it was observed that aerenchyma levels were low in internodes that were elongating; however, 7–10 days after internodes reached full elongation, higher levels of aerenchyma were observed in *D*‐genotypes. This indicated that stem aerenchyma formation is suppressed in nascent and growing internodes of sorghum stems and that the induction of aerenchyma formation occurs following internode elongation. If *SbNAC_D* is involved in stem aerenchyma formation, then expression of Sobic.006G147400 may increase following internode elongation in parallel with the formation of stem aerenchyma. To test this possibility, internodes from the three pairs of RILs with contrasting *D*‐locus/*d1*‐locus haplotypes were collected when they first reached full length (time point 1, low aerenchyma) and again 7–10 days later (time point 2) when higher levels of aerenchyma were visible in genotypes encoding *SbNAC_D* (but not in *SbNAC_d1*). The results showed that Sobic.006G147400 expression was significantly higher in internodes that were forming stem aerenchyma (time point 2) in *SbNAC_D* genotypes (Figure 5). In addition, increased expression of Sobic.006G147400 was also observed 7–10 days after internode elongation in *SbNAC_d1* genotypes even though aerenchyma levels did not increase in these genotypes. This indicates that increased expression of Sobic.006G147400 was induced by development and not caused by the formation of aerenchyma. To complete the survey of gene expression, RNA extracted from the internodes from one pair of RILs (RIL49, *SbNACd1*; RIL392, *SbNAC_D*) was used to analyze expression of the seven other genes in the *D*‐locus by qRT‐PCR (Supporting Information Table S4). Only one of these genes, Sobic.006g148000, showed an increase in expression in stems between time point 1 and time point 2 in RIL392 (*SbNAC_D*) (Supporting Information Table S4). However, no deleterious coding sequence variants were identified in the parental lines used to fine‐map *D*‐locus QTL (Supporting Information Table S3), suggesting that Sobic.006G148000 plays some other role in internode development.

The developmental timing of *SbNAC_D* expression and aerenchyma accumulation in sorghum stems was examined in more detail during vegetative phase stem growth using R07020, a tall, photoperiod‐sensitive, late‐flowering, high‐biomass genotype. Growth of R07020 in 14 hr day‐lengths represses floral initiation allowing analysis of stem aerenchyma formation during an extended period of vegetative growth after completion of the juvenile phase. R07020 internode elongation begins between ~45 and 55 days after germination, and aerenchyma were observed in fully elongated internodes produced during vegetative development. Qualitative visual inspection of cross sections showed that aerenchyma were also present in the leaf midribs and leaf sheaths of R07020 that developed during the vegetative phase.

**Figure 6:**
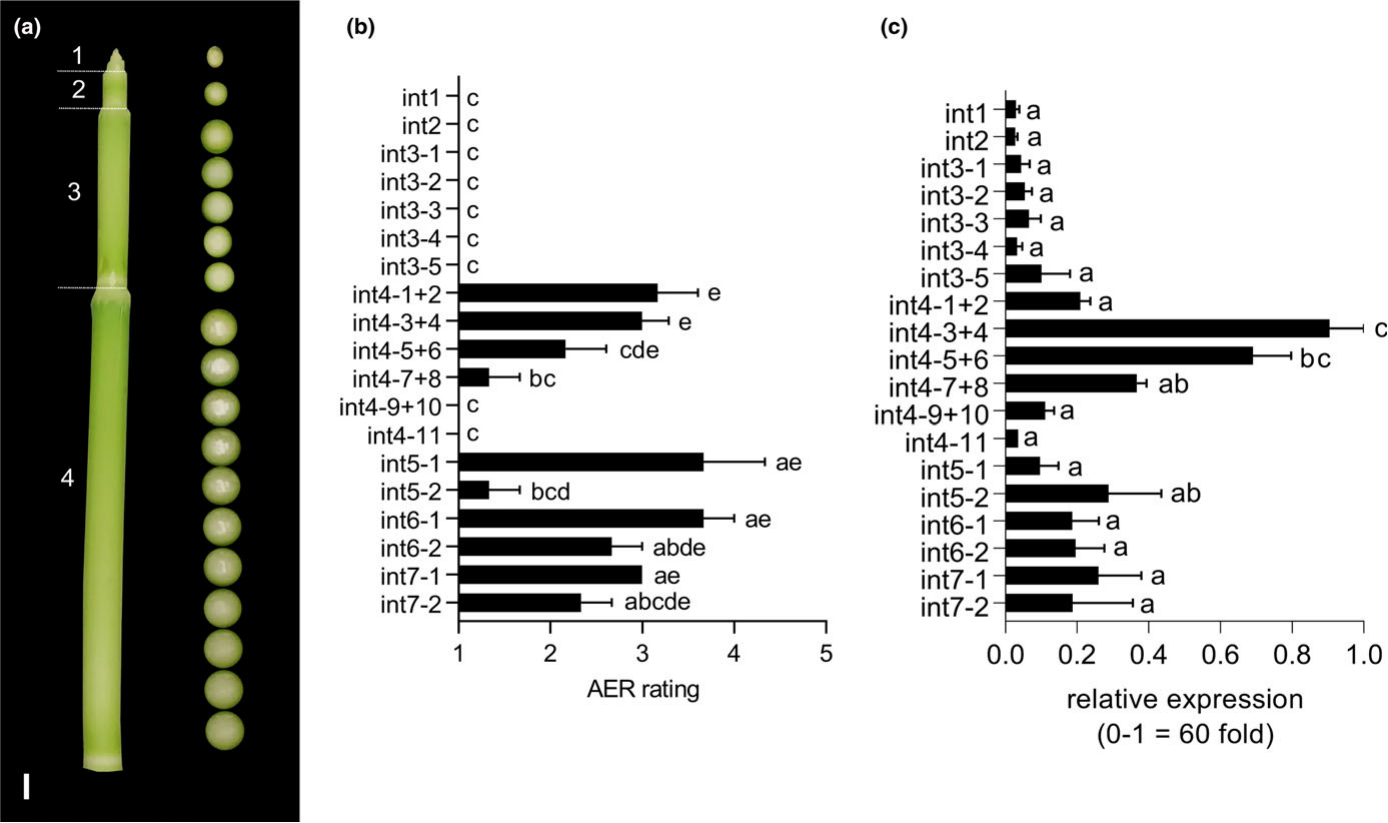
Aerenchyma (AER) formation and expression of *SbNAC_D* in stem sections of R07020. (a) Photograph of the top of a R07020 stem. Internode numbers are indicated to the left of the stem. R07020 remained in the vegetative phase throughout these experiments, and internode 1 is the youngest visible internode below the apical dome. Photographs of cross sections of each internode taken every 1 cm are shown to the right of the stem in (a). No AER is visible in internodes 1–3 or in the lower portion of internode 4. AER formation begins in the top of internode 4 in the center of the internode. AER forms in a basipetal manner and spreads radially outward. Scale bar = 1 cm. (b) AER was rated on a scale from 1 (no AER) to 5 (high AER). Ratings of pairs of adjacent sections of internode 4 (int4) were averaged. AER formation of internodes 5–7 was rated 2 cm from the top node of each internode (int5‐1, int6‐1, int7‐1) and 2 cm from the bottom node of each internode (int5‐2, int6‐2, int7‐2). Each rating is the average of five biological replicates. Tukey's multiple comparison test was used to compare ratings of each internode section. Sections with different letters are significantly different from each other, *p* < 0.05. (c) Relative expression of *SbNAC_D* across internode sections of R07020. Expression of *SbNAC_D* is correlated with AER formation (Spearman's *r* = 0.709). Relative expression was calculated via the $2^{-\Delta \Delta C_{t}}$ method relative to the internode section with the highest expression (Pfaffl, 2001). Expression values are the average of three biological replicates. Fold change in expression between the minimum and maximum values on the *y*‐axis was calculated based on SbUBC normalized values according to $\text{FC}=2^{\Delta C_{t}\left(\max\right)-\Delta C_{t}\left(\min\right)}$

The timing of aerenchyma formation during growth and development of stem internodes was examined using cross‐sectional analysis of R07020 stems (Figure 6a). The stems of rapidly growing R07020 plants contained two short nascent internodes immediately below the shoot apex (Int1, Int2), followed by an elongating internode (Int3) and several older fully elongated internodes (Int4‐7) (Figure 6a, left). The two nascent nonelongated internodes (Int1, Int2) and the partially elongated internode 3 contained very low levels of aerenchyma (Figure 6b). Aerenchyma were present in the upper end of fully elongated internode 4 (sections Int4‐1 to Int4‐6) but not at the basal end of this internode (Int4‐9 to Int4‐11). Aerenchyma were observed throughout older internodes (Int5‐7), although at lower abundance near the base of internode 5 (Figure 6a,b).

Expression of *SbNAC_D* during internode development in R07020 was analyzed using qRT‐PCR (Figure 6c). Stem internode sections used for expression analysis were identical to those used for analysis of aerenchyma accumulation (Figure 6b). *SbNAC_D* was expressed at relatively low levels in the two youngest nonelongated internodes located immediately below the shoot apex that lacked aerenchyma (Int1, Int2) (Figure 6c). *SbNAC_D* expression increased to a small extent in internode 3 and reached maximal levels in the upper portion of internode 4 (Figure 6c, sections Int4‐1 + 2 to Int4‐7 + 8), a region where aerenchyma were visible (Figure 6, Int4). Aerenchyma levels and *SbNAC_D* expression were low at the basal end of internode 4 (i.e., Int4, sections Int4‐9−Int4‐11). *SbNAC_D* expression was also observed in tissue of older internodes (Int5‐7). There was a strong correlation between *SbNAC_D* expression and aerenchyma levels during vegetative stem internode development (*ρ* = 0.709).

### 3.7 SbXCP1 expression is correlated with aerenchyma formation and regulated by *SbNAC_D*

**Figure 7:**
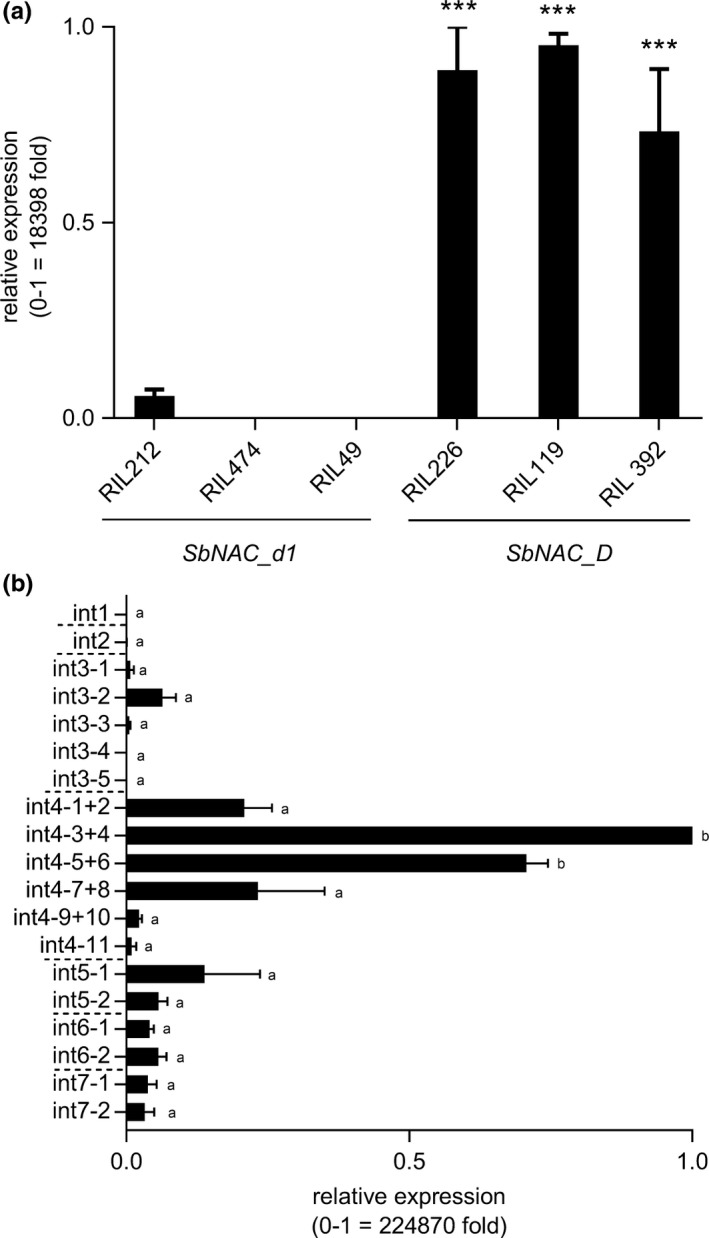
Expression of *SbXCP1* in (a) SbNAC_d1/SbNAC_D RILs and (b) R07020 internodes. *SbXCP1* is a developmental programmed cell death (PCD) marker gene. (a) The expression of *SbXCP1* is elevated in *SbNAC_D* RILs (RIL119, RIL392, RIL226) compared to *SbNAC_d1* RILs (RIL212, RIL474, RIL49). The uppermost internode of each RIL was sampled at a time point after aerenchyma (AER) formation had started. Expression differences between RILs were compared by Tukey's multiple comparison test, *p* < 0.05. (b) Expression of *SbXCP1* is low in internode sections where no AER formation is occurring (int1, int2, int3‐X). Expression increases in sections of internode 4 (int4‐X) where AER are forming. Tukey's multiple comparison test was used to compare ratings of each internode section. Sections with different letters are significantly different from each other, *p* < 0.05. All gene expression was normalized to the expression of *SbUBC* in each sample. Relative expression was calculated via the $2^{-\Delta \Delta C_{t}}$ method relative to the internode section with the highest expression of each gene (Pfaffl et al., 2001). Fold change in expression between the minimum and maximum values on the *y*‐axis was calculated based on *SbUBC* normalized values according to $\text{FC}=2^{\Delta C_{t}\left(\max\right)-\Delta C_{t}\left(\min\right)}$. Expression values are the average of three biological replicates

The formation of stem aerenchyma could be mediated by programmed cell death (PCD). Induction of cysteine proteases is associated with PCD in rice (Yin & Xue, 2012) and has been correlated with developmental PCD in Arabidopsis (Olvera‐Carillo 2015). Therefore, we identified the sorghum homolog (Sobic.007g172100, *SbXCP1*) of a rice PCD‐regulated gene (Os02g48450) that encodes a cysteine protease and characterized its expression to see whether it would be a useful marker for sorghum stem aerenchyma formation. RNA extracted from internodes of the pairs of RILs encoding either *SbNAC_D* or *SbNAC_d1* (RIL392/RIL49, RIL474/RIL119, RIL226/RIL212) was used for qRT‐PCR analysis of *SbXCP1* gene expression. In the RILs that encode *SbNAC_D*,* SbXCP1* expression increased in parallel with appearance of aerenchyma in internodes (Figure 7a, right). However, *SbXCP1* expression was not significantly induced in RILs that encode *SbNAC_d1* (Figure 7a, left), indicating that *SbNAC_D* is required for induction of *SbXCP1* expression in stems during aerenchyma formation.

The correlation between internode development, aerenchyma formation, and expression of the PCD marker gene *SbXCP1* was also investigated during R07020 stem development (Figure 7b). The relative expression of *SbXCP1* was low in internodes 1 and 2, with a small increase in internode 3, and was high in the mid‐to‐upper portion of internode 4 (Int4, sections 3–8) (Figure 7b). Elevated but lower expression was observed in internodes 5, 6, and 7. In general, variation in the expression of the PCD marker gene *SbXCP1* was similar to *SbNAC_D* expression and matched the time course and location of aerenchyma formation in stems during internode development. Expression of six of the other genes in the delimited *D*‐locus was also analyzed during internode development in R07020 (Supporting Information Figure S7).

### 3.8 Spatial analysis of aerenchyma accumulation in sorghum internodes

**Figure 8:**
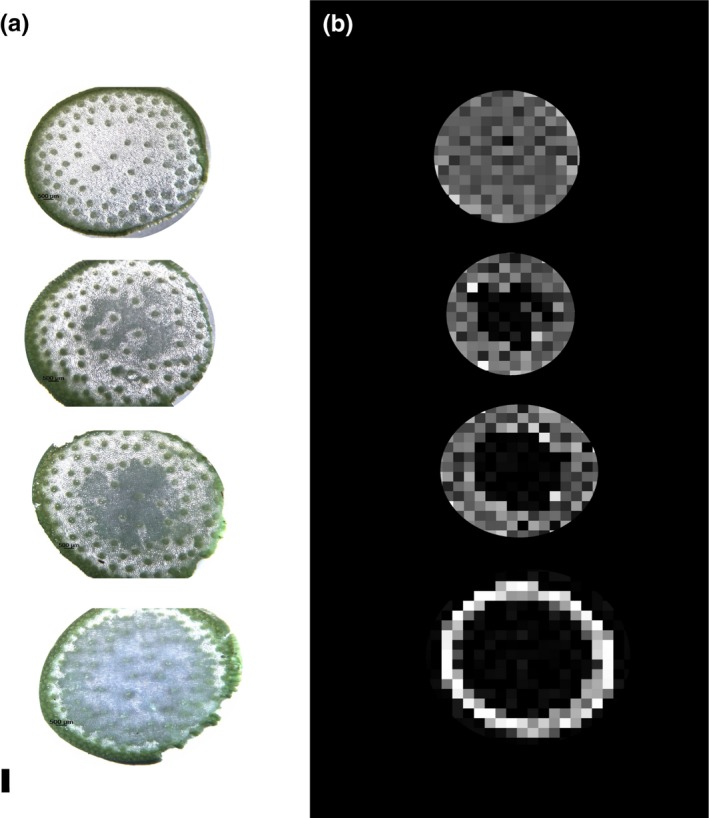
Microscopic (a) and CT scan (b) images of R07020 stem cross sections at progressive stages of aerenchyma (AER) formation. From the top down, solid stem with no AER formation to high levels of AER formation across the stem cross section. AER formation begins in the pith parenchyma cells at the center of the stem and spreads radially outward toward the rind. Scale bar = 0.1 cm

Microscopic analysis of internode cross sections and CT analysis showed that aerenchyma form first in pith parenchyma located between vascular bundles (VBs) in the center of the internode (Figure 8). As internode development proceeds, aerenchyma accumulation spreads radially, with relative few aerenchyma forming in the rind (Figure 8). Pith parenchyma cells located between VBs were initially converted into aerenchyma leaving islands of VBs and closely associated cells (Figure 8). At later stages of internode development, nearly all of the cells surrounding VBs located in the central region of the stem had been converted into aerenchyma (Figure 8).

## 4 DISCUSSION

Genotypic variation in sorghum stem aerenchyma formation was initially documented in 1916 (Hilson, 1916). This genetic determinant of variation in stem/leaf midrib aerenchyma accumulation was named the *D*‐locus and further characterized in subsequent studies (Ayyangar et al., 1936; Stephens & Quinby, 1939; Swanson & Parker, 1931). Sorghum genotypes with opaque white leaf midribs were reported to have drier, “pithy” stems, whereas genotypes with green midribs had stems with low levels of aerenchyma that accumulated higher amounts of sugars postflowering. More recent research identified QTL that modulate sorghum leaf midrib color and stem moisture content (Burks et al., 2015; Guan et al., 2011; Han et al., 2015; Hart et al., 2001; Mocoeur et al., 2015; Srinivas et al., 2009; Xiao‐ping et al., 2011; Xu et al., 2000). In the current study, a main effect QTL on SBI06 was identified in three populations that have a large impact on the accumulation of stem aerenchyma. This QTL aligned with a leaf midrib color QTL previously designated as the *D*‐locus (“*D*” for dry stems) (Ayyangar et al., 1936; Hart et al., 2001; Xu et al., 2000). QTL‐assisted candidate gene identification and analysis of candidate gene coding variants and gene expression identified Sobic.006g147400, a gene encoding a NAC transcription factor, as the best candidate for the *D*‐gene (*SbNAC_D*).

Numerous important sorghum genes involved in fertility restoration, flowering time, seed shattering, and the regulation of stem growth have been cloned using QTL‐assisted map‐based gene identification (Hilley et al., 2016, 2017; Klein et al., 2005; Lin et al., 2012; Murphy et al., 2011, 2014). This well‐established approach was used in the current study to identify a gene and alleles that could explain variation in phenotypes associated with the *D*‐locus. QTL that modulate the extent of stem aerenchyma accumulation were mapped in three sorghum populations derived from parents that differed in stem aerenchyma ratings. A single, large‐effect QTL on SBI06 was identified in all three populations. Genetic analysis showed that BTx623, the female parent of each population, was the source of sequence variation that reduced aerenchyma accumulation in stems. Fine‐mapping delimited the *D*‐locus to 73kbp, a region encoding eight genes. The sequences of the eight genes in the delimited *D*‐locus derived from the four parental lines used for QTL analysis were examined to identify sequence variants that could affect protein‐coding regions. This survey identified a disruptive sequence variant in one of the eight genes in BTx623 that was not present in the other parental lines used for QTL analysis. The key variant introduced a stop codon in the coding region of Sobic.006G147400 resulting in truncation of the NAC transcription factor encoded by this gene. No other disruptive coding variants were identified in BTx623 in genes in the *D*‐locus. Disruptive mutations were not present in the coding regions of four of the genes in the delimited *D*‐locus, and disruptive coding variants found in the other three genes were present in one or two of the parental lines crossed to BTx623 (IS3620c, R07007, Standard broomcorn) but not all three genotypes. Based on the analysis of coding variants, Sobic.006G147400 was tentatively identified as a candidate for the *D*‐gene and the two alleles were named *SbNAC_D* and *SbNAC_d1*. The association between *SbNAC_D* genotype and stem aerenchyma phenotype was extended by screening a panel of diverse sorghum genotypes for *SbNAC_D* alleles and stem aerenchyma accumulation. This analysis showed that genotypes encoding *SbNAC_D* had higher levels of stem aerenchyma relative to genotypes encoding *SbNAC_d1*. Further evidence for the involvement of *SbNAC_D* in the formation of stem aerenchyma was obtained by analyzing the expression of *SbNAC_D* during stages of internode development before and after the onset of aerenchyma accumulation. Analysis of the internode located immediately below the peduncle in plants at flag leaf stage showed that *SbNAC_D* expression was low prior to the accumulation of aerenchyma and much higher 7–10 days later when internodes were accumulating aerenchyma. The correlation between increases in *SbNAC_D* expression and onset of aerenchyma formation was also high in vegetative phase internodes of R07020. In contrast, the expression of the other seven genes located in the delimited *D*‐locus showed lower or no correlation with stem aerenchyma formation compared to *SbNAC_D*. In addition, expression of a PCD marker gene encoding a cysteine protease (*SbXCP1*) was highly correlated with expression of *SbNAC_D* and the accumulation of aerenchyma in stems and expression was not induced in stems of genotypes encoding *SbNAC_d1*. The involvement of a NAC transcription factor in stem aerenchyma formation is not surprising since NAC transcription factors have been associated with other types of PCD. For example, the NAC transcription factors VND6/7 play key roles in vascular tissue differentiation, a developmental process that includes PCD (Escamez & Tuominen, 2014). Similarly, NAC transcription factors mediate PCD that occurs during senescence (Kim et al., 2014; Uauy et al., 2006), root cap development (ANAC033) (Fendrych et al., 2014), and PCD associated with oxidative stress (ANAC13) (De Clercq et al., 2013). Phylogenetic analysis showed that the grass homologs of *SbNAC_D* formed a cluster that was differentiated from clusters of NAC transcription factors involved in secondary cell wall formation and senescence. Taken together, these results are consistent with the identification of *SbNAC_D* (Sobic.006g147400) as the *D*‐gene, a major modulator of aerenchyma formation in stems.

Sorghum genotypes vary in the timing and extent of aerenchyma formation in leaf midribs and stems during development (Hilson, 1916). In the current study, aerenchyma were not detected in nonelongated nascent internodes of vegetative phase plants and the onset of aerenchyma accumulation was associated with completion of internode elongation. Intercalary meristems and zones of cell elongation are located at the base of elongating internodes in sorghum (Kebrom et al., 2017). In vegetative phase plants, aerenchyma were first observed in the more fully developed upper portion of recently elongated internodes. As internode development proceeds, aerenchyma spread radially from the center of the stem toward the rind/epidermis and in a basipetal manner until most of the internode was filled with aerenchyma. The stem nodes generally lacked or had low levels of aerenchyma throughout development. Rice shows a similar pattern of aerenchyma accumulation during stem and internode development (Steffens, Geske, & Sauter, 2011). The timing of expression of *SbNAC_D* during stem and internode development was consistent with the gene's proposed role in stem aerenchyma formation. *SbNAC_D* was expressed at very low levels in the top two nascent internodes, internode growing zones, and nodes of developing internodes, tissues that accumulate only low levels of aerenchyma. *SbNAC_D* expression increased in the upper portion of fully expanded internodes in the same location where aerenchyma form first during development. Moreover, analysis of internodes derived from *SbNAC_D* and *SbNAC‐d1* RILs that differ in the extent of stem aerenchyma formation differentially expressed a gene encoding a sorghum homolog of a cysteine protease similar to vignain (SbXCP1) that is involved in developmental programmed cell death in rice (Yin & Xue, 2012). The expression of this gene increased in parallel with *SbNAC_D* expression and the appearance of stem aerenchyma in stem internodes during development. In contrast, expression of *SbXCP1* remained low in internodes of a sorghum genotype encoding *SbNAC_d1,* indicating that *SbNAC_D* regulates the expression of *SbXCP1* directly or indirectly. Taken together, these results are consistent with the identification of *SbNAC_D* (Sobic.006g147400) as the *D*‐gene, a major modulator of aerenchyma formation in stems.

Programmed cell death plays an important role in plant development in response to pathogens and abiotic stress (Kabbage, Kessens, Bartholomay, & Williams, 2017; Van Hautegem, Waters, Goodrich, & Nowack, 2015). The accumulation of aerenchyma in stem pith parenchyma cells provides a way to remobilize nutrients from these cells to support rapid growth of stems and leaves during the vegetative phase. This may be one reason aerenchyma formation is correlated with rapid stem elongation during the vegetative phase. The selective destruction of pith parenchyma cells located in the stem also reduces maintenance respiration. However, the destruction of these stem cells decreases the capacity of stems for sucrose storage that occurs in some genotypes when plants transition to the reproductive phase. By anthesis, the demand for nutrients to support stem and leaf growth has declined and excess sugars accumulate in the vacuoles of pith parenchyma stem cells. A large supply of stem sugars is useful during the grain filling phase, especially as a buffer during periods of water deficit and other stresses that inhibit photosynthesis.

The recessive *SbNAC_d1* allele was present in Blackhull Kafir, Hegari, and Collier, genotypes that were imported from Africa into the United States between 1881 and 1908 (Smith & Frederiksen, 2000). This indicates that the *SbNAC_d1* allele was originated in Africa and was introduced at the onset of sorghum use in the United States consistent with the wide distribution of this allele in U.S. sorghum germplasm (Smith & Frederiksen, 2000). Following introduction into the United States, Blackhull Kafir (*SbNAC_d1*) was used in the development of lines such as BTx3197 (*SbNAC_d1*) and BTx623 (*SbNAC_d1*), which are useful for grain sorghum hybrid production. SC170, a genotype used in breeding R‐lines such as RTx430, also encodes *SbNAC_d1*. Therefore, the historically important grain hybrid derived by crossing ATx623*RTx430 was homozygous for *SbNAC_d1*. The identification of *SbNAC_d1* in genotypes used for grain sorghum breeding suggests that reduced levels of stem aerenchyma are beneficial in these crops, possibly by reducing lodging or by increasing stem sugars that could provide a source of carbohydrate for grain filling under conditions of drought or other adverse environmental conditions.

The sweet sorghum genotypes Rio, Della, Wray, Collier, and Grassl all encode *SbNAC_d1* alleles, and the stems of these genotypes had lower levels of aerenchyma compared to genotypes encoding *SbNAC_D*. Reducing stem aerenchyma is important in sweet sorghum because retention of stem pith parenchyma increases the capacity for sucrose accumulation in the large vacuoles of these cells. Two previous sorghum genetic studies that characterized QTL for stem juiciness and sugar yield did not identify QTL aligned with *SbNAC_D* (Felderhoff et al., 2012; Murray et al., 2008). The parental genotypes used in these studies (BTx623*Rio, BTx3197*Rio) all encode *SbNAC_d1* consistent with lack of stem moisture or sugar content QTL aligned with the *D*‐locus (Felderhoff et al., 2012; Murray et al., 2008). These previous studies identified other QTL that affect stem sugar content, concentration, and juiciness, indicating that the genetic basis of these traits is complex (Felderhoff et al., 2012; Murray et al., 2008).

Analysis of *NAC_D* homologs in other grasses showed that in the genotypes of wheat and sugarcane, analyzed *NAC_D* homologs contained large deletions suggesting that breeders may have selected for inactive versions of *NAC_D* in these species as occurred in sorghum. In sugarcane, selection for inactive alleles of *NAC_D* is consistent with low levels of stem aerenchyma, high stem juiciness, and high stem sucrose content in that crop (Waclawovsky, Sato, Lembke, Moore, & Souza, 2010). Selection for more solid stems has also occurred in wheat in part to increase resistance to insects that damage stems although the genetic basis of insect resistance is complex (Nilsen et al., 2017).

The identification of alleles of *SbNAC_D* will make marker‐assisted selection or genome editing of sorghum genotypes for various end uses easier, although screening for white/green leaf midribs as an indicator of *D*‐gene activity is reliable and easy to implement. More importantly, the identification of *SbNAC_D* provides an entry point for the identification of factors that regulate the tissue‐specific expression of *SbNAC_D* during development and genes regulated by *SbNAC_D* that modulate aerenchyma formation. Variation in the extent of stem aerenchyma formation was observed in genotypes that encode *SbNAC_D*, and low levels of variation in stem “pithiness” were observed in populations derived from parental lines containing *SbNAC‐d1* (Felderhoff et al., 2012). Follow‐up studies are needed to more fully elucidate the factors that regulate *SbNAC_D* expression, the developmental PCD pathway that leads to stem aerenchyma formation, and other genes that modulate stem and leaf midrib aerenchyma formation.

## CONFLICT OF INTEREST

The authors have no conflict of interests to declare.

## AUTHOR CONTRIBUTIONS

A.L.C. and J.M. designed experiments and wrote the article; A.L.C. performed most of the experiments; B.M. performed RNAseq data analysis; K.M.J.Y. and A.L.C. performed DNA sequencing; W.L.R. provided seeds and populations; and J.M. supervised experimental design and article preparation.
