# Supplementary material for: Sorghum stem aerenchyma formation is regulated by SbNAC_D during internode development
Source: Plant Direct. 2018 Nov 12;2(11):e00085. doi: 10.1002/pld3.85 (PMC6508845; doi:10.1002/pld3.85)
Supplement: Supplementary file 1 [file PLD3-2-e00085-s001.pdf]

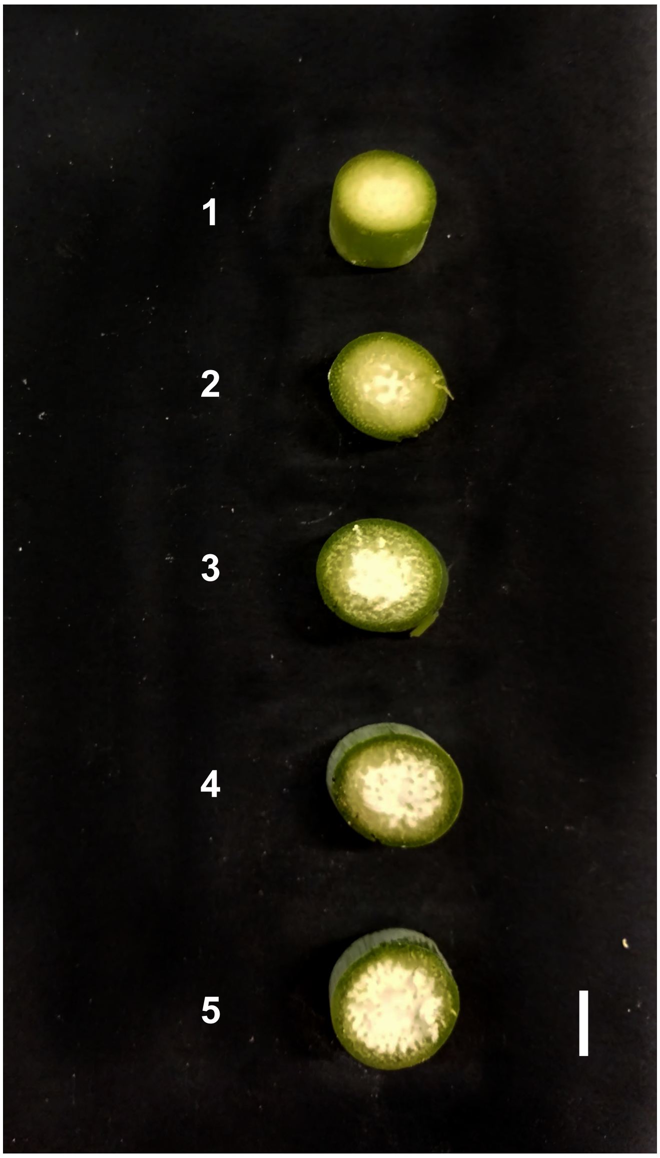

Supplemental figure 1. Stem aerenchyma rating scale, 1 (no aerenchyma) to 5 (high aerenchyma)

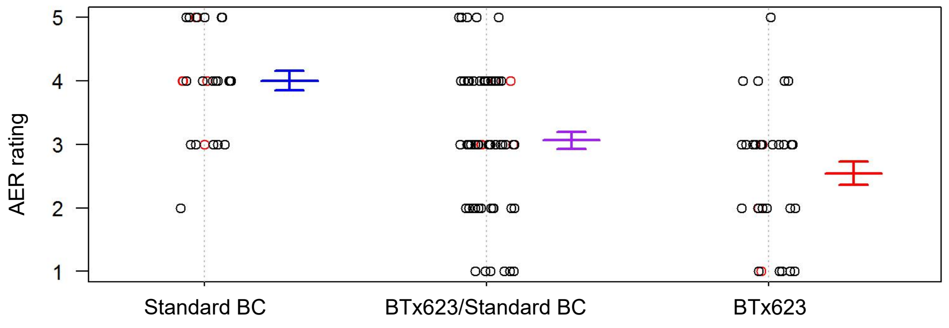

Supplemental figure 2. Phenotype-genotype plot of stem aerenchyma ratings in the F2 popultion BTx623*Standard Broomcorn at the marker with the highest LOD score in the QTL interval. The open circles represent AER ratings of individuals in the population. The average AER ratings of individuals genotyped as Standard BC at this marker were higher than the average rating of individuals genotyped as BTx623. Heterozygous individuals had intermediate phenotypes.

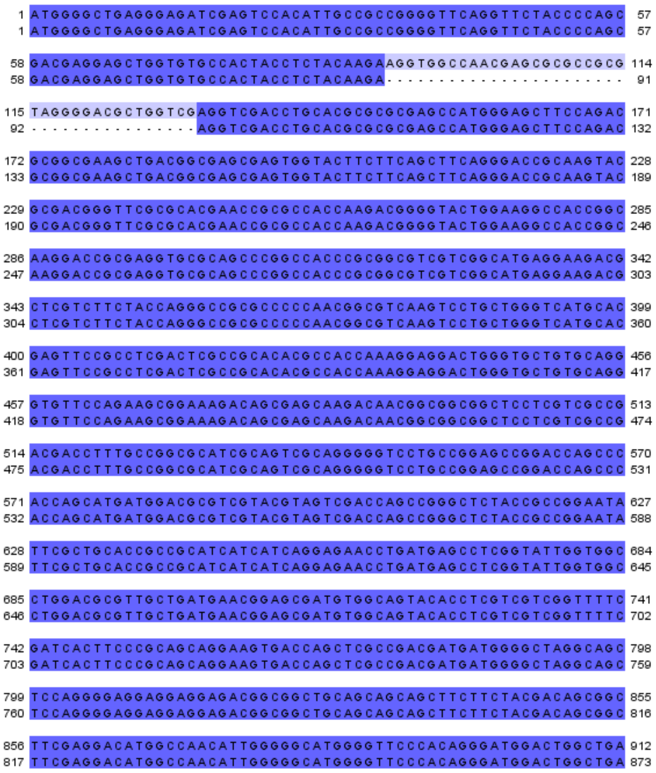

Supplemental figure 3. Transcript sequences of the candidate gene Sobic.006G147400 obtained from Sanger sequencing (top) and from Phytozome (bottom). The first exon of the Sobic.006G147400 transcript sequence available on Phytozome contains a 39 nucleotide deletion because those bases were misannoted as an intron, likely because of the stop codon mutation (at 115 nt) in BTx623.

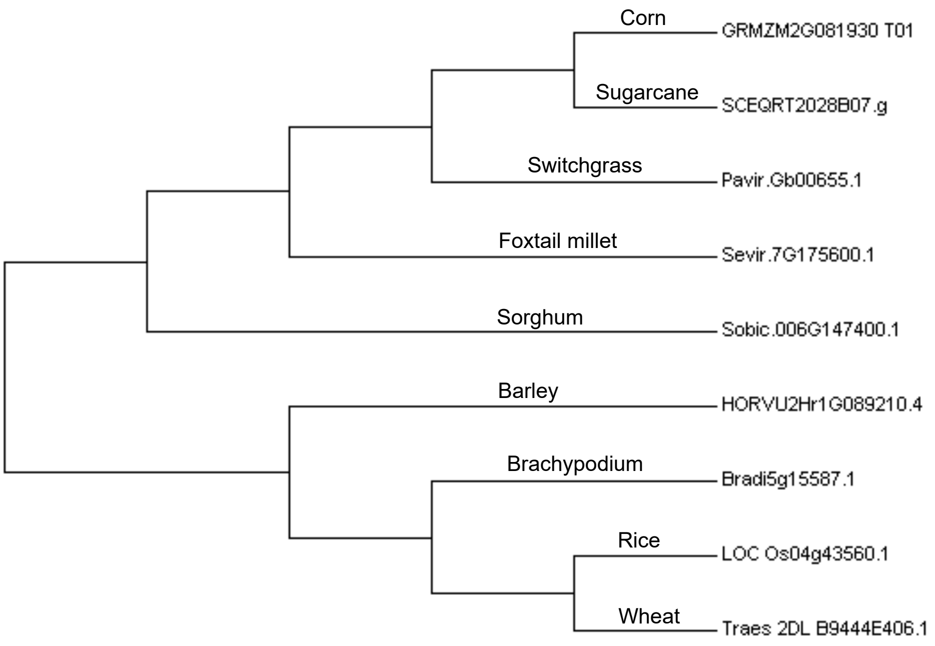

Supplemental figure 4. Phylogenetic analysis of grass homologs of SbNAC_D. Unroote phylogenetic analysis grouped SbNAC_D homolgs into C4 (i.e. sorghum, corn) and C3 (i.e. barley, rice) grasses. Relationships were inferred using hte Neighbor-Joining method (Jones et al., 1992). All positions containing gaps and missing data were eliminiated. Evolutionary analyses were conducted in MEGA7 (Kumar et al., 2016).

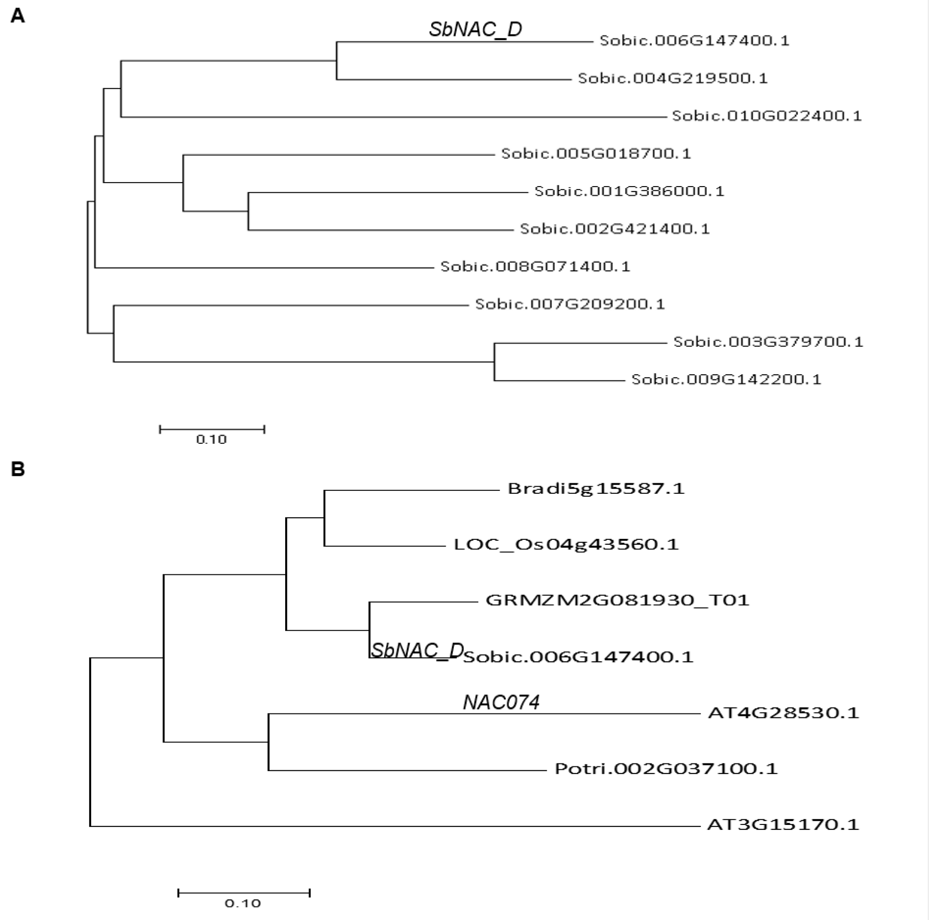

Supplemental figure 5. Phylogenetic relationships of SbNAC_D to (A) SbNAC_D paralogs in the sorghum genome and (B) SbNAC_D orthologs in other plant species. Relationships were inferred using the Neighbor-Joining method (Jones et al., 1992). All positions containing gaps and missing data were eliminated. Evolutionary analyses were conducted in MEGA7 (Kumar et al., 2016).

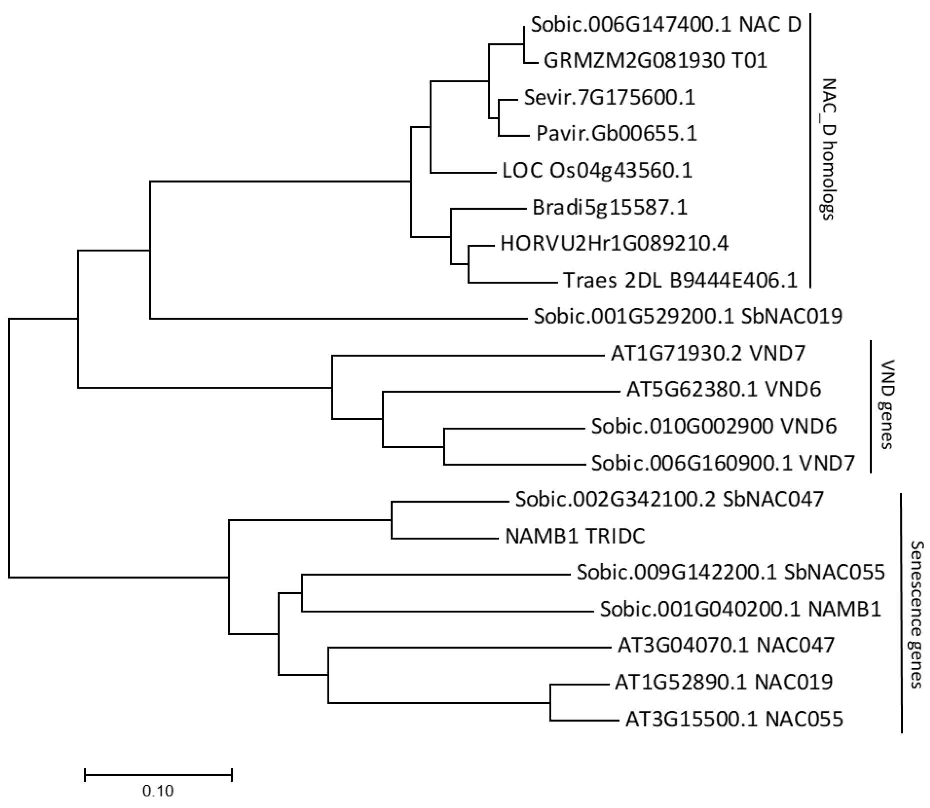

Supplemental figure 6. Phylogenetic relationships of SbNAC_D homologs in grasses to NAC-family VND genes involved in vascular differentiation and NAC-family genes involved in senescence. Relationships were inferred using the Neighbor-Joining method (Jones et al, 1992). All positions containing gaps and missing data were eliminated. Evolutionary analyses were conducted in MEGA7 (Kumar et al, 2016).

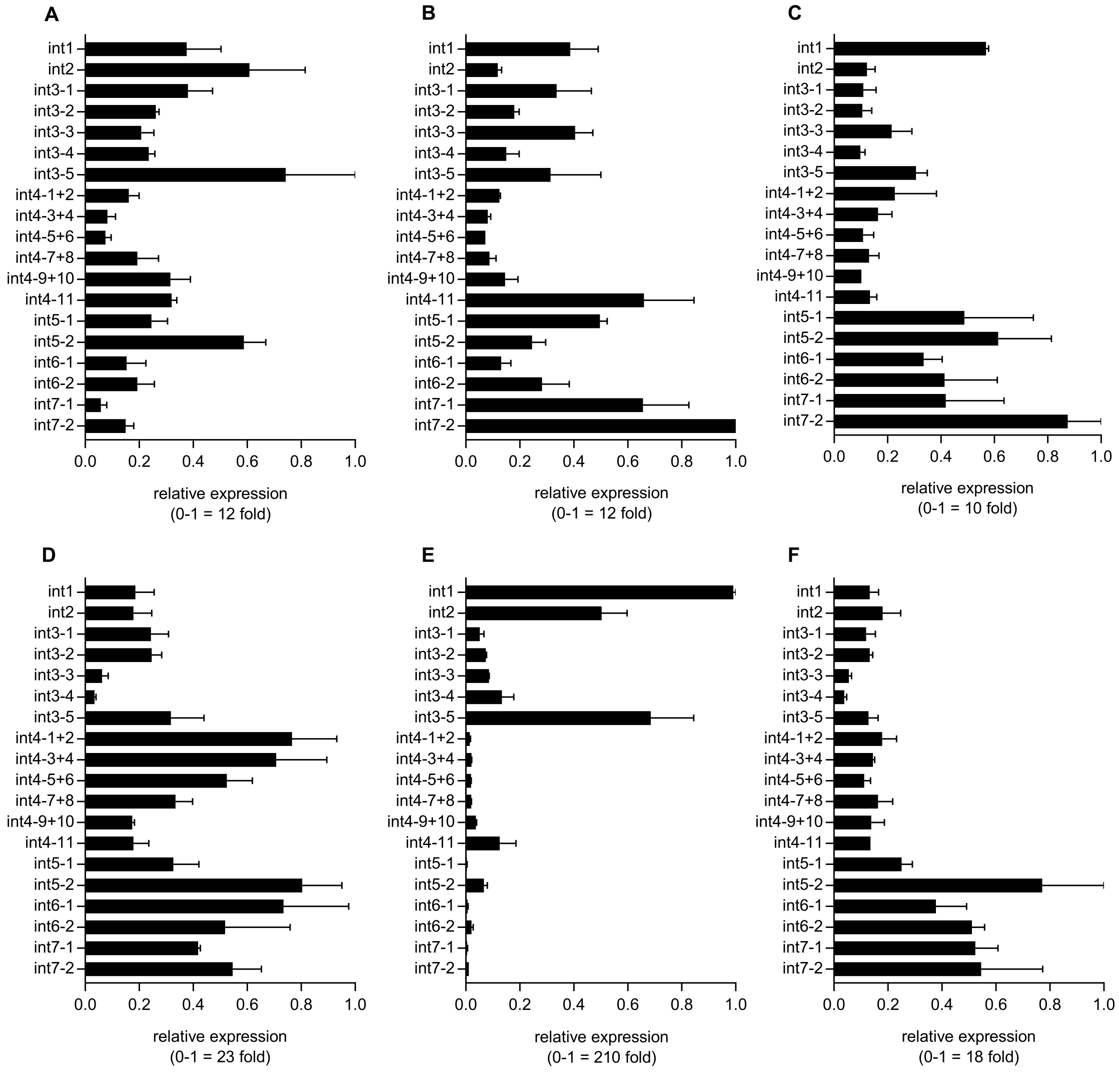

Supplemental figure 7. Expression of genes in the NAC_D QTL interval during the development of internodes of R07020 stems: (A) Sobic.006g147450, (B) Sobic.006g147500, (C) Sobic.006g147600, (D) Sobic.006g147800, (E) Sobic.006g147900, (F) Sobic.006g148000. Internodes were numbered from the apex of the plant downward. Whole internodes were collected from the top two nacent, unelongated internodes below the shoot apex (int1, int2). The next internodes (int3) was elongating and was sectioned into five 1-cm sections from the top down (int3-X). Internode 4 was also divided into 1-cm sections (int4-X). Adjacent pairs of sections from internode 4 were averaged for simplicity and since their expression values were very similar. From internode 5 (int5), internode 6 (int6), and internode 7 (int7), the top 2 cm and the bottom 2 cm of each internode were collected. Expression of the genes in the NAC_D QTL interval was not well correlated with AER formation. Relative expression was calculated via the $2^{\wedge -\Delta\Delta Ct}$ method relative to the internode section with the highest expression (Pfaffl et al., 2004). Expression values are the average of 3 biological replicates. Fold change in expression between the minimum and maximum values on the y axis were calculated based on SbUBC normalized values according to FC = $2^{\Delta Ct(max)-\Delta Ct(min)}$. No expression could be detected from Sobic.006g147700 in R07020 tissues.

Supplemental Table 1. 2 LOD QTL intervals from three biparental mapping populations

Population	CHR	2-LOD interval (CHR_Mb)	Peak position (Mb)	LOD score
BTx623/IS3620c	6	6_50820292 - 6_51044545	50.8	30.1
BTx623/R07007	6	6_48273018 - 6_51329068	50.8	43.6
BTx623/Std Broomcorn	6	6_50954277 - 6_51787450	51.5	7.37

Supplemental Table 2. Genes in the fine-mapped QTL region

Gene name	Description	Location
Sobic.006G147400	No apical meristem (NAM) protein (NAM)	Chr06:50896169..50898604 forward
Sobic.006G147450	Threonine aldolase	Chr06:50902561..50905501 forward
Sobic.006G147500	Predicted protein	Chr06:50914667..50916048 forward
Sobic.006G147600	Threonine aldolase	Chr06:50922630..50925976 reverse
Sobic.006G147700	Threonine aldolase	Chr06:50929529..50933978 reverse
Sobic.006G147800	Predicted protein	Chr06:50941894..50945361 forward
Sobic.006G147900	Predicted protein	Chr06:50948145..50951111 forward
Sobic.006G148000	Myb protein	Chr06:50959448..50962420 reverse

Supplemental Table 3. Coding region sequence variants in genes in the NAC_D QTL interval among the parental lines used in QTL mapping. Deleterious alleles are highlighted in red. Variant calls were taken from publicly available whole genome sequence data (Phytozome v12.1.6). The effect of each amino acid change was determined by PROVEAN analysis (Choi et al. 2012).

Gene ID	Position (Chr_Mb)	BTx623	IS3620c	R07007	Standard Broomcorn	Rio	SC170	Amino acid change	Effect
Sobic.006G147400	6_50896283	**T**	C	C	C	**T**	**T**	X39Q	Deleterious
Sobic.006G147450	6_50903023	A	A	A	C	C	C	T95P	Neutral
Sobic.006G147500									No variants
Sobic.006G147600	6_50924009	A	**G**	A	A	A	**G**	S218P	Deleterious
Sobic.006G147600	6_50924916	G	G	T	T	G	T	P128Q	Neutral
Sobic.006G147700	6_50931496	T	T	T	**A**	T	T	K267I	Deleterious
Sobic.006G147700	6_50932111	G	G	G	C	G	G	Q164E	Neutral
Sobic.006G147700	6_50932356	G	G	G	C	G	G	L122V	Neutral
Sobic.006G147700	6_50932467	C	C	C	**G**	C	C	F293L	Deleterious
Sobic.006G147800	6_50944184	T	C	C	C	C	C	F208S	Neutral
Sobic.006G147800	6_50944189	G	A	A	A	A	A	A210T	Neutral
Sobic.006G147800	6_50944209	A	C	C	C	C	C	L216F	Neutral
Sobic.006G147800	6_50944295	T	T	**C**	**C**	**C**	**C**	I245T	Deleterious
Sobic.006G147800	6_50944541	C	C	C	C	A	A	T327L	Neutral
Sobic.006G147800	6_50944760	T	T	**A**	**A**	**A**	**A**	V400D	Deleterious
Sobic.006G147800	6_50944922	T	T	C	C	C	C	L454S	Neutral
Sobic.006G147800	6_50945034	C	C	A	A	A	A	S491R	Neutral
Sobic.006G147800	6_50945071	C	C	**A**	**A**	C	**A**	P504T	Deleterious
Sobic.006G147900	6_50948305	G	G	C	C	G	C	A11P	Neutral
Sobic.006G147900	6_50948336	T	T	C	C	T	C	F21S	Neutral
Sobic.006G147900	6_50948375	T	T	C	C	T	C	V32A	Neutral
Sobic.006G147900	6_50948380	G	G	T	T	G	T	A36S	Neutral
Sobic.006G147900	6_50948386	G	G	T	T	G	T	A38S	Neutral
Sobic.006G147900	6_50950605	C	C	G	G	C	G	L111V	Neutral
Sobic.006G147900	6_50950719	A	A	G	G	G	G	N149D	Neutral
Sobic.006G147900	6_50950813	C	C	C	C	C	**T**	S180F	Deleterious
Sobic.006G148000	6_50960848	T	G	T	G	G	G	Y238S	Neutral

Supplemental Table 4. Expression genes in the D-locus in the internode subtending the peduncle was analyzed during development in RIL49 (NAC-d1) and RIL392 (NAC_D). RNA was extracted from the internode from RIL49 and RIL392 prior to aerenchyma formation (TP1) and 7-10 days later (TP2) when aerenchyma were visible. Gene expression was analyzed by qRT-PCR and are reported as fold-induction.

Gene ID	RIL	NAC_D allele	Fold change in expression between TP1 – TP2	p-value (NS, not significant)
Sobic.006G147400	RIL49	*SbNAC_d1*	25	$p < 0.05$
Sobic.006G147400	RIL392	*SbNAC_D*	1010	$p < 0.05$
Sobic.006G147450	RIL49	*SbNAC_d1*	-	NS
Sobic.006G147450	RIL392	*SbNAC_D*	-	NS
Sobic.006G147500	RIL49	*SbNAC_d1*	-	NS
Sobic.006G147500	RIL392	*SbNAC_D*	-	NS
Sobic.006G147600	RIL49	*SbNAC_d1*	-	NS
Sobic.006G147600	RIL392	*SbNAC_D*	-	NS
Sobic.006G147700	RIL49	*SbNAC_d1*	-	NS
Sobic.006G147700	RIL392	*SbNAC_D*	-	NS
Sobic.006G147800	RIL49	*SbNAC_d1*	-	NS
Sobic.006G147800	RIL392	*SbNAC_D*	-	NS
Sobic.006G147900	RIL49	*SbNAC_d1*	-	NS
Sobic.006G147900	RIL392	*SbNAC_D*	-	NS
Sobic.006G148000	RIL49	*SbNAC_d1*	-	NS
Sobic.006G148000	RIL392	*SbNAC_D*	8	$p < 0.005$

Supplemental Table 5. Sobic.006G147400 DNA and cDNA sequencing primers

Type	Genotype	Forward primer (5' → 3')	Reverse primer (5' → 3')
DNA template	IS3620c, Tx2910, BTx3297, RTx2909, Rio, BTx623	CAGCAGCGGTTTCTTTTGCT	TCAGCTGAACAGTCAGAAACCTT
DNA template	Tx7000	CCTGGCATGACACTACAGCA	AGTCAGCTGAACAGTCAGAAAC
DNA template	All remaining genotypes	CAGGCTGCAAGAGCGAGATA	TCGATCAGTCCTCTCAGCCA
DNA Sequencing	-	CCTGCAGGCTTCCTCGTCCCTATAAA	GCTCGTTGGCCACCTTCTTGTAGAGG
DNA Sequencing	-	CCTCTACAAGAAGGTGGCCAACGAGC	CAGCCCTCTGCACCATGTGATGCAC
DNA Sequencing	-	GTGCATCACATGGTGCAGAGGGCTG	GGTGACGAGTCTTGTGCGTTGGGATC
DNA Sequencing	-	GATCCCAACGCACAAGACTCGTCACC	AAGACGAGCGTCTTCCTCATGCC
DNA Sequencing	-	CCGCATCATCATCAGGAGAACCTG	CAGGTTCTCCTGATGATGATGCGG
DNA Sequencing	-	GGCATGAGGAAGACGCTCGTCTT	CCTTTTGGATTGGAGAGGTGCACTC
cDNA template	BTx623, IS3620c	CAGGCTGCAAGAGCGAGATA	CCTTCACAATGCCCAGTCCA
cDNA sequencing	-	CAGGCTGCAAGAGCGAGATA	TCTTTCCGCTTCTGGAACAC
cDNA sequencing	-	GTGTGCCACTACCTCTACAAG	ACATCGCTCCGTTCATCAG
cDNA sequencing	-	CGAGTGGTACTTCTTCAGCTTC	CCTTCACAATGCCCAGTCCA

Supplemental Table 6. qPCR primers

Gene ID	gene name	Forward primer (5' → 3')	Reverse primer (5' → 3')
Sobic.006G147400	*SbNAC_D*	AGAGTGCACCTCTCCAATCC	GCAAATGAAAATGACACCTCCT
Sobic.007G172100	*SbXCP1*	GTGAAGAACTCGTGGGGACC	ATGCGATTCAGAGCTCGTCG
Sobic.001G526600	*SbUBC*	CATGCTGCACATTCGCATAG	AGAGACATGGTCCACAAGAAC
